# Supplementary material for: Evaluation of NADES for Pectin Films Reinforced with Oxalic Acid-Modified Chitin Nanowhiskers
Source: Polymers (Basel). 2025 Feb 21;17(5):572. doi: 10.3390/polym17050572 (PMC11902133; doi:10.3390/polym17050572)
Supplement: Supplementary file 1 [file polymers-17-00572-s001.zip › polymers-3458965-supplementary.pdf]

Article

Evaluation of NADES for Pectin Films Reinforced with Oxalic Acid-Modified Chitin Nanowhiskers

Andrea Mathilde Mebert [1,2], Cynthia Melisa Melian-Queirolo [1,2], María Fernanda Hamet [3], Guillermo Javier Copello [1,2] and Andrea Gomez-Zavaglia [3,*]

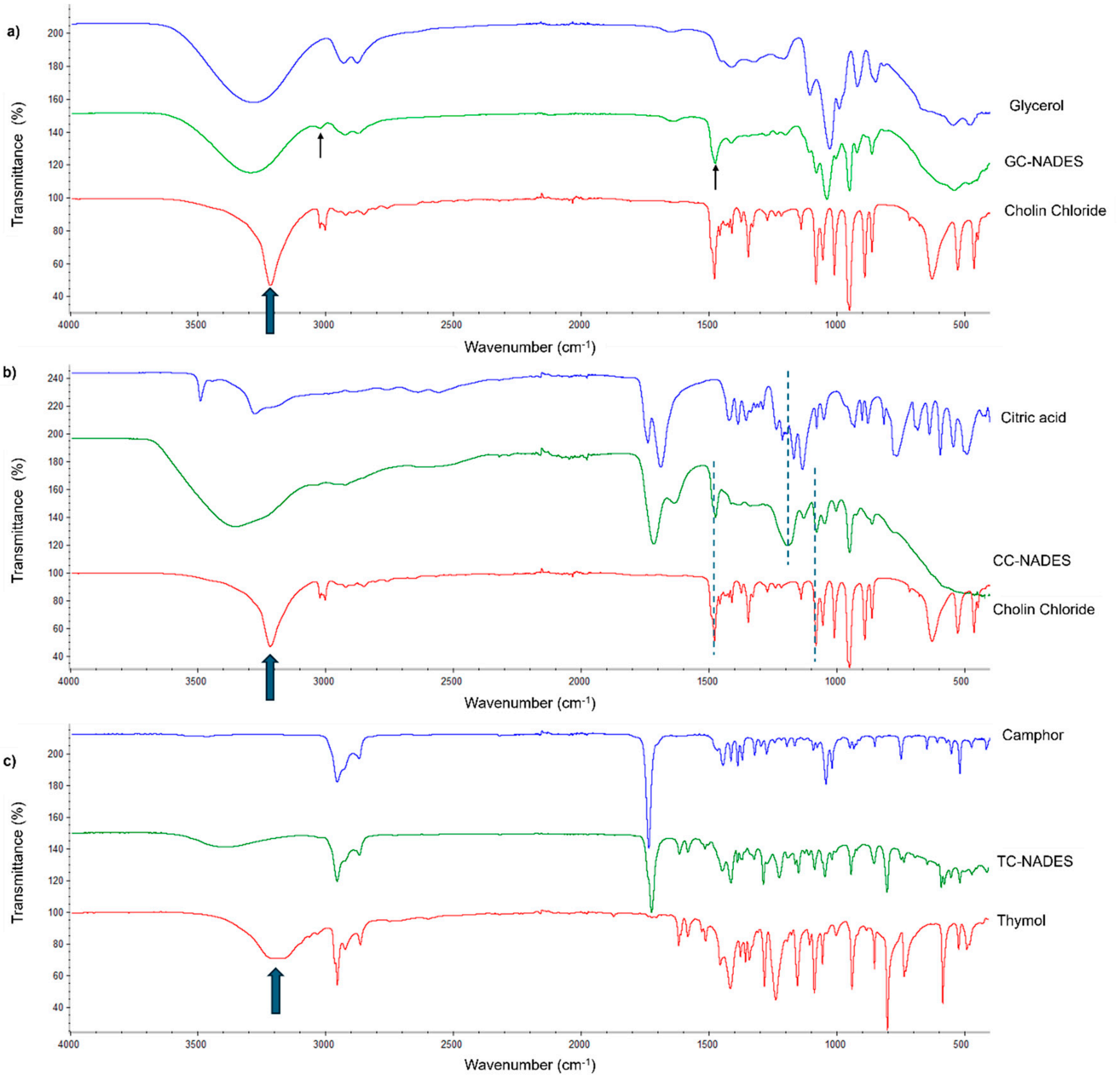

Figure S1. FT-IR Spectra of HBD (blue), NADES (green) and HBA (red) of (**a**) GC-NADES (**b**) CC-NADES (**c**) TC-NADES.

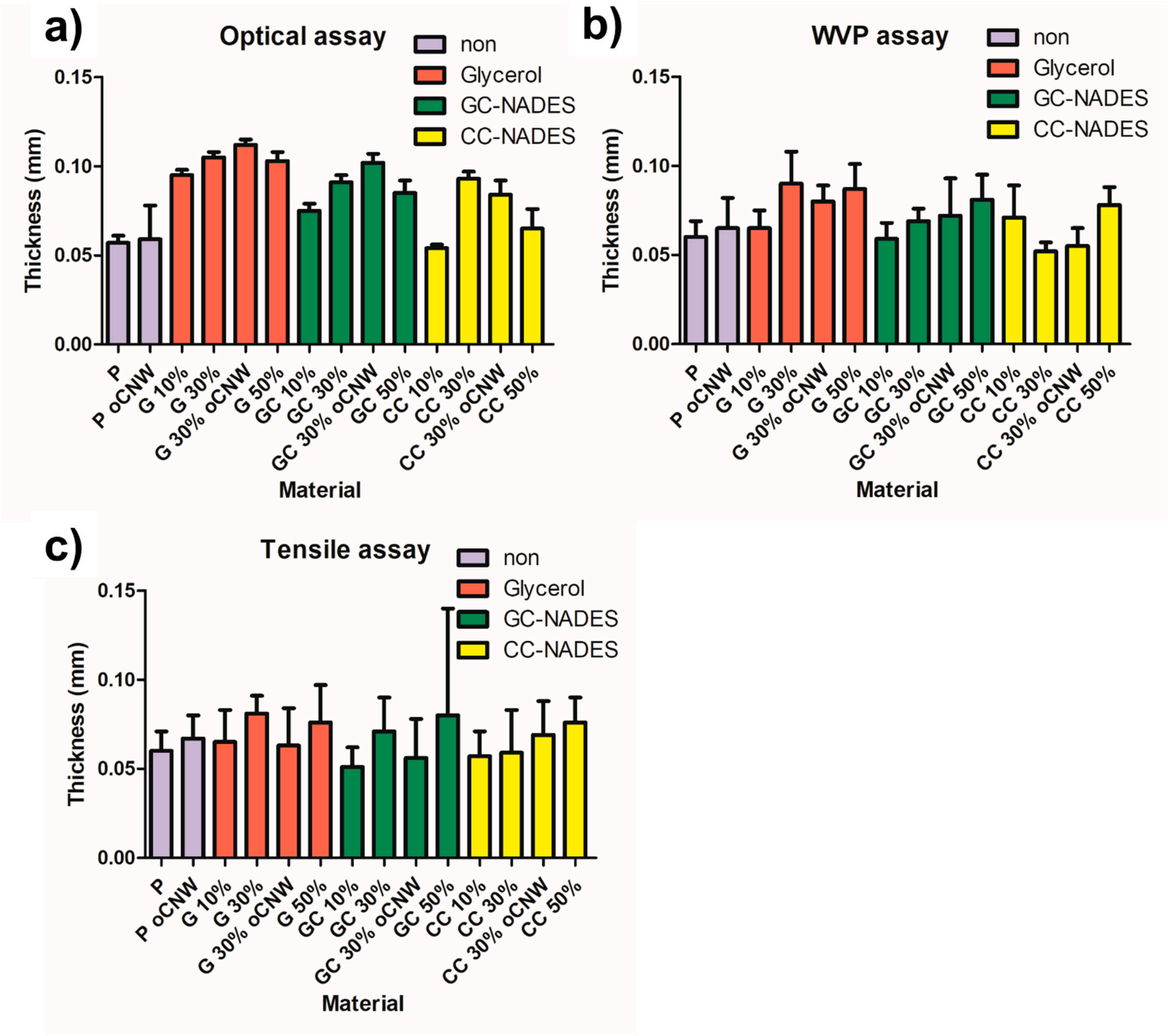

Figure S2. Thickness in mm of non (blue) plasticized, and glycerol (red), GC- (green) and CC-NADES (yellow) plasticized films of (**a**) Opacity (**b**) WVP (**c**) Traction assays.

Table S1. Thickness of different films Optical properties, WVP and tensile assay.

	Thickness (mm)					
Material	Opacity assay		WVP assay		Traction Assay	
	Mean	SD	Mean	SD	Mean	SD
P	0.057	0.004	0.060	0.009	0.060	0.011
P oCNW	0.059	0.019	0.065	0.017	0.067	0.013
G 10%	0.095	0.003	0.065	0.010	0.065	0.018
G 30%	0.105	0.003	0.090	0.018	0.081	0.010
G 30%oCNW	0.112	0.003	0.080	0.009	0.063	0.021
G 50%	0.103	0.005	0.087	0.014	0.076	0.021
GC 10%	0.075	0.004	0.059	0.009	0.051	0.011
GC 30%	0.091	0.004	0.069	0.007	0.071	0.019
GC 30% oCNW	0.102	0.005	0.072	0.021	0.056	0.022
GC 50%	0.085	0.007	0.081	0.014	0.080	0.060
CC 10%	0.054	0.002	0.071	0.018	0.057	0.014

CC 30%	0.093	0.004	0.052	0.005	0.059	0.024
CC 30% oCNW	0.084	0.008	0.055	0.010	0.069	0.019
C 50%	0.065	0.011	0.078	0.010	0.076	0.014

Table S2. Transparency as %T or %T/*X* and Opacity as Abs or Abs/X at 600 nm.

	**%T**		**%T/mm**		**Abs**		**Abs/mm**	
**Material**	**Mean**	**SD**	**Mean**	**SD**	**Mean**	**SD**	**Mean**	**SD**
P	82.7	0.5	1460.5	8.35	0.083	0.002	1.461	0.044
P oCNW	80.9	0.1	1374.7	1.70	0.092	0.001	1.564	0.009
G 10%	79.0	0.4	1460.5	8.35	0.083	0.002	1.081	0.022
G 30%	80.2	0.1	761.2	0.55	0.096	0.000	0.907	0.003
G 30% oCNW	78.1	1.3	1460.5	8.35	0.083	0.002	0.961	0.065
G 50%	84.6	1.9	822.5	17.98	0.073	0.010	0.705	0.093
GC 10%	82.2	0.6	1460.5	8.35	0.083	0.002	1.139	0.040
GC 30%	81.8	0.2	895.9	1.90	0.087	0.001	0.956	0.010
GC 30% oCNW	74.4	3.6	1460.5	8.35	0.083	0.002	1.262	0.207
GC 50%	81.2	0.4	955.7	4.45	0.090	0.002	1.062	0.024
CC 10%	82.8	0.8	1460.5	8.35	0.083	0.002	1.517	0.075
CC 30%	78.0	1.1	840.2	11.52	0.108	0.006	1.165	0.064
CC 30% oCNW	79.7	2.1	1460.5	8.35	0.083	0.002	1.176	0.135
CC 50	76.3	1.1	1167.7	16.24	0.118	0.006	1.804	0.093

Table S3. WVP values of different films.

	**WVP (g/msPa)**	
**Material**	**Mean**	**SD**
P	0.203	0.027
P oCNW	0.206	0.007
G 10%	0.198	0.011
G 30%	0.388	0.001
G 30% oCNW	0.334	0.010
G 50%	0.398	0.010
GC 10%	0.192	0.002
GC 30%	0.330	0.046
GC 30% oCNW	0.311	0.033
GC 50%	0.377	0.033
CC 10%	0.193	0.011
CC 30%	0.163	0.009
CC 30% oCNW	0.147	0.008
CC 50%	0.278	0.049

Table S4. Tensile properties of the films.Tensile strength (σm), Young's modulus (E), and elongation at break (εB).

	**σm (MPa)**		**E (MPa)**		**εB (%)**	
**Material**	**Mean**	**SD**	**Mean**	**SD**	**Mean**	**SD**
P	33.6	8.1	7.9	1.7	2.7	1.5
G 10%	27.8	5.8	6.9	2.8	4.8	0.9
G 30%	33.4	10.4	6.6	3	10.7	4.9
G 50%	17.7	6	2.7	1.1	17.1	10.1
G 30% oCNW	34.1	4.3	9.5	2.1	6.8	2
GC 30%	36.3	10.2	6.3	1.5	8.3	8

GC 50%	23.4	7.3	5	1.6	7.3	2.8
GC 30% oCNW	36.2	7.6	10	1.8	9.9	3.4
CC 10%	41.2	15.9	17.7	6	3.2	1.1
CC 30%	49.6	4.1	11.4	3.2	4.6	3.4
CC 50%	31.8	5.6	6.4	1.1	10.6	4.9
CC 30% oCNW	45	17.9	14.8	5.2	4.8	2.6

Table S5. Summarized results as % change compared to pure pectin films. Significant changes are indicated with *. Water Vapor Permeability (WVP), Tensile strength (σm), Young's modulus (E), and elongation at break (εB).

	% change Respecting to Pure Pectin Film					
**Material**	**Transparency**	**Opacity**	**WVP**	**σm**	**E**	**εB**
P	0	0	0	0	0	0
P oCNW	−2	7	1	--	--	--
G 10%	−4	−26 ***	−2	−17	−13	78
G 30%	−3	−38 ***	91 ***	−1	−16	296
G 50%	2	−52 ***	96 ***	−47	−66	533 ***
G 30% oCNW	−6 *	−34 ***	65 ***	1	20	152
GC 10%	−1	−22 **	−5	--	--	--
GC 30%	−1	−35 ***	63 ***	8	−20	207
GC 50%	−2	−27 ***	86 ***	−30	−37	170
GC 30% oCNW	−10 ***	−14	53 **	8	27	267
CC 10%	0	4	−5	23	124 ***	19
CC 30%	−6 *	−20 **	−20	48	44	70
CC 50%	−8 **	23 *	37 *	−5	−19	293
CC 30% oCNW	−4	−20 **	−28	34	87	78

The statistical analysis was performed by one-way ANOVA followed by Tukey's post-test, where *, ** and *** indicate statistical significant differences with $p < 0.05$, p < 0.01 and $p < 0.001$ respectively.
